# Multi-Sensor Monitoring of Underground Heritage Sites: A Case Study of the Dragon’s Den Cave and St. Kinga’s Chapel in Poland

**DOI:** 10.3390/s26196329

**Published:** 2026-10-07

**Authors:** Mateusz Jabłoński, Wojciech Jaśkowski

**Affiliations:** AGH University of Krakow, Faculty of Geo-Data Science, Geodesy, and Environmental Engineering, Al. A. Mickiewicza 30, 30-059 Krakow, Poland; jaskow@agh.edu.pl

**Keywords:** structural health monitoring, underground heritage, telemetric monitoring, multi-sensor system, displacement monitoring, strain monitoring, microclimate, geodetic verification, early warning, salt mine

## Abstract

**Highlights:**

**What are the main findings?**

- An integrated telemetric monitoring system combined crack meters, a borehole extensometer, rock-bolt load cells, and microclimate sensors in two underground UNESCO heritage sites.
- Long-term observations confirmed stable structural behavior under humid and saline conditions, with no significant deformation trends.

**What are the implications of the main findings?**

- Temperature monitoring and repeated measurements are essential for distinguishing environmental and technical effects from genuine structural changes and avoiding false alarms.
- Continuous local sensing, periodic geodetic verification, and terrestrial laser scanning provide complementary information for minimally invasive preventive conservation and safety management.

**Abstract:**

Long-term structural health monitoring of underground heritage sites is challenging because of high humidity, limited accessibility, complex geological conditions, and aggressive saline environments. This study presents the implementation and long-term assessment of an integrated multi-sensor telemetric monitoring system deployed in two historically significant sites: Dragon’s Den Cave beneath Wawel Castle and St. Kinga’s Chapel in the Wieliczka Salt Mine. The system combines displacement sensors, strain measurements on rock anchors, and microclimate monitoring to assess structural behavior and support the early detection of potentially hazardous changes. Monitoring was conducted for four years in the cave and two years in the salt mine. Structural measurements were analyzed together with environmental data to distinguish temperature- and humidity-related sensor responses from actual deformation. Periodic geodetic surveys provided independent verification of the telemetric measurements. Both structures remained stable throughout the observation periods. Telemetric measurements recorded changes of up to 0.16 mm at the monitored extensometer locations, while geodetic surveys indicated cumulative displacements of up to approximately 2.6 mm horizontally and 2.4 mm vertically. The results demonstrate that integrating structural and environmental sensing with periodic geodetic verification enables reliable long-term monitoring under demanding underground conditions. The proposed minimally invasive and scalable approach supports preventive conservation and safety management of underground heritage structures.

## 1. Introduction

For many decades, miners, surveyors, geologists, geotechnical engineers, and site administrators have undertaken a wide range of measures aimed at protecting historic underground structures from degradation [1]. These activities are focused on preserving either the climatic conditions of a given site or its geometry, which together determine the overall safety of the structure.

Preservation of climatic conditions should be understood as maintaining an environment conducive to the development or survival of naturally occurring fauna and flora. Such tasks are usually carried out by specialists in the biological sciences, often supported by engineering solutions related to ventilation systems and geotechnical or mining support measures.

Geometric changes, understood as structural deformations—such as convergence or separation of roofs and sidewalls—are identified by multidisciplinary teams of specialists representing the above-mentioned fields [2]. These activities include detailed inventory surveys of the underground structure, periodic deformation measurements, geomechanical analyses performed on the basis of precise numerical models, the preparation of specialized protection designs, and the implementation of these protective measures [3,4]. Support measures implemented both in active mining operations and in historic mines as well as underground structures of natural origin can be divided into four main categories [5]:

- roof and sidewall bolting while maintaining an open excavation space;
- construction of excavation linings;
- installation of timber cribs over a designated area (this option also occurs in a combined form with rock bolting);
- complete liquidation through backfilling of the underground void.

Such support methods may induce additional stresses within the supported chamber, either redistributed into zones different from the original stress field or as increased unit stresses in the surrounding rock mass. Consequently, they may influence adjacent underground voids or excavations.

A significant challenge in the deformation analysis of a supported chamber is its frequent geometric alteration resulting from scaling operations and adjustments of the excavation shape to accommodate the support system itself. Therefore, prior to the implementation of any support works, it is essential to acquire baseline reference data for subsequent deformation monitoring of the chamber’s surroundings, taking into account the design of the planned support system [6].

The basis for conducting deformation monitoring of any structure, and underground structures in particular, is an inventory survey of the object with a level of detail appropriate to the scope of the study, combined with an initial (baseline) measurement of the monitoring network [7]. This does not always require a complete inventory of the entire structure; in many cases, surveying a polygonal traverse, convergence bases, or fracture aperture widths may be sufficient; however, for large and complex multi-level underground systems, adjacent voids and chambers secured in the recent past may have a significant influence on the protected structure [8].

This issue is particularly relevant for structures exposed to water-related hazards, such as salt mines and karst caves. A representative example is the “Wieliczka” Salt Mine, where archival maps are systematically processed and accessible workings are subjected to laser scanning to produce a three-dimensional model. This approach enables more accurate geomechanical analyses and the design of appropriate mining support systems. The mine represents an extremely complex system of chambers excavated using various techniques since the 13th century; nevertheless, such an approach to inventory surveying and geological recognition of the surroundings of a protected underground structure is applicable in any case.

Analyses of underground loading conditions in castles or fortifications, identification of voids surrounding natural karst caves, or determination of the size and spatial arrangement of chambers and galleries in military or post-mining structures must be based on comprehensive geodetic inventory surveys [9]. At present, the most accurate method for such inventory surveys is laser scanning, performed in either terrestrial or mobile form [10,11]. There are also methodologies that combine laser scanning measurements with photogrammetric techniques, further enhancing the completeness and accuracy of spatial documentation [12,13].

It should be noted that the accuracy of laser scanning, even when referenced to a control network and tie points, does not allow for precise determination of structural deformations. It may be assumed that the error of a single point within a point cloud, for a hanging traverse extending over several hundred meters, may reach up to 1 cm. When analyzing deformation based on successive measurement campaigns, this error should be treated as a root mean square value, resulting in deformation detection accuracy on the order of up to 2 cm.

Consequently, classical geodetic methods—while providing only point-based information—offer higher accuracy for deformation analysis. These include polygonal traverse surveys conducted in at least two measurement series and executed using the multi-setup method, supplemented by gyroscopic azimuth determination in areas most distant from reference points [14], as well as independent measurements of benchmarks and control points using precise leveling performed in closed loops.

In practice, such surveys allow vertical deformation accuracy on the order of 0.3 mm per kilometer and horizontal deformation accuracy of approximately 2 mm per kilometer of traverse length. While these results are highly valuable, measurements of this type must be conducted over extended time intervals in order for deformation processes to develop and become detectable. Depending on the intensity of the phenomenon, observation periods of approximately 0.5 to 5 years are required to reliably identify deformation trends [15].

As a separate category of deformation studies, the measurement of fractures and convergence—i.e., local deformations—should be considered. Although these measurements are also performed periodically, they are carried out in a manner that allows for identical repetition of the measurement procedure at clearly defined reference points. Using instruments such as calipers, convergence meters, measuring rods, or distance meters, convergence bases can be measured with significantly higher accuracy. Depending on the applied measurement method, the accuracy of determining such bases may range from micrometers to approximately 1–2 mm. For deformation monitoring understood in this local sense, laser scanning may be applied under appropriate conditions to obtain more spatially distributed results, although with lower accuracy than classical point-based measurements [8].

Recent developments in structural health monitoring have increasingly focused on continuous data acquisition, automatic data transmission, remote access to measurement results, automated detection of changes and alarm conditions, and the integration of heterogeneous information sources [16,17]. These capabilities extend the possibilities of traditional periodic inspections and isolated measurements by enabling the systematic observation of structural responses over time. Robotic and automated inspection platforms may further improve access to underground and otherwise difficult-to-reach structures, while reducing the need for personnel to work in potentially hazardous environments [16]. The adoption of robotic technologies for structural health monitoring is influenced by several non-technical factors, including accessibility, safety, regulatory support, organizational readiness, and data-driven decision-making [18]. These considerations are particularly important in protected historic underground sites, where the use of robotic or automated platforms must comply with conservation requirements and the physical constraints of the monitored environment.

Recent studies of underground engineering systems emphasize that reliable assessment should account for the interaction of multiple thermal, hydraulic, pressure-related, and mechanical parameters rather than rely on a single indicator [19,20]. Although these studies concern gas hydrate production and enhanced geothermal reservoirs, respectively, their methodological conclusions are relevant to underground heritage monitoring. In such environments, temperature-dependent sensor responses, moisture-related effects, local discontinuities, and structural deformation should be considered jointly when assessing long-term stability. This supports the use of complementary monitoring techniques rather than reliance on a single measurement system.

A solution corresponding to the requirements described above is the installation of sensor arrays that perform continuous or quasi-continuous measurements of geometric changes at predefined measurement bases, depending on the objectives and requirements of the monitoring system [21,22]. The first group includes instruments designed to measure changes in crack width, such as crack meters based either on changes in electrical resistance or on vibrating wire technology. Other sensors commonly used for monitoring historic underground structures include anchor load cells and extensometers installed in boreholes to detect roof and sidewall separation [23]. The interpretation of such measurements should also account for the heterogeneous nature of underground media and the possible influence of pre-existing discontinuities, which may cause deformation to develop locally and non-uniformly [24].

For this reason, continuous telemetric monitoring should not be considered a substitute for independent spatial measurement techniques. Local sensor observations may identify changes at specific points, but they do not fully describe the spatial distribution or geometric context of deformation. In the present study, telemetric measurements were therefore complemented by periodic geodetic surveys and terrestrial laser scanning. This combined approach enabled both the continuous observation of selected structural parameters and the periodic verification of the overall geometric condition of the monitored historic underground structures. The integration of these complementary methods also reduced the risk of interpreting localized sensor responses as representative of the behavior of the entire structure.

### Research Aim

This study addresses the continuous and quasi-continuous monitoring of two underground heritage sites in southern Poland: the karst Dragon’s Den Cave beneath Wawel Hill in Kraków and the salt-carved St. Kinga’s Chapel in the Wieliczka Salt Mine. Both sites form part of nationally and internationally recognized cultural heritage and are open to large numbers of visitors. Their structural condition may be affected by slow geological and geotechnical processes, including convergence, fracture widening, and rheological creep, as well as by changes in the underground microclimate. These factors may threaten both the structural integrity of the sites and the preservation of their historic and artistic fabric.

The aim of this research is to apply and evaluate an integrated monitoring approach that combines high-resolution telemetric sensors—including crack meters, bolt load cells, and downhole extensometers—with periodic geodetic measurements, such as traverse surveys, precise leveling, and terrestrial laser scanning, together with microclimatic observations. The approach is intended to:

- detect and quantify active deformation processes at a resolution relevant to conservation decision-making;
- support early identification of conditions potentially relevant to visitor safety and heritage protection; and
- provide actionable information for conservators, engineers, and site managers in planning interventions, maintenance, and visitor management measures while minimizing the visual and physical impact on the fabric of these historic underground spaces.

The originality of this study is methodological rather than instrumental. The individual monitoring techniques used—local deformation sensors, load cells, extensometers, environmental sensors, geodetic surveys, and terrestrial laser scanning—are established measurement methods. The contribution of the present work lies in their coordinated integration into a site-adapted monitoring framework for underground heritage structures. Continuous or quasi-continuous point-based measurements are combined with periodic spatial observations, allowing local sensor responses to be assessed against independent geometric and geodetic control. Environmental observations are interpreted jointly with mechanical measurements to help distinguish potentially reversible microclimatic effects from persistent structural changes. The framework is evaluated in two underground heritage sites with different geological settings, geometries, functions, and conservation constraints.

## 2. Materials and Methods

As case studies, the authors selected two sites included on the UNESCO World Heritage List: the Dragon’s Den Cave located beneath the Castle Hill of Wawel in Kraków, Poland and the Chapel of St. Kinga situated in the Wieliczka Salt Mine located in Wieliczka, Poland. At both sites, modern sensors designed for quasi-continuous monitoring have been installed. In addition, classical geodetic measurements are carried out, including polygonal traverse surveys with distributed point measurements, precise leveling of benchmarks, periodic laser scanning, and convergence measurements [25].

The Dragon’s Den Cave is one of the most recognizable caves in Poland and is listed as an immovable historic monument. Furthermore, it is located within the Wawel Hill, which is inscribed on the UNESCO World Heritage List [26]. The cave is visited by more than 300,000 people annually. It is classified as a Class 0 object, meaning that its technical condition requires particularly strict supervision. As a karst cave burdened by numerous historic structures and located directly adjacent to the Vistula River, the Dragon’s Den Cave is exposed to the effects of natural karst processes, convergence of the roof and sidewalls, and detachment of rock blocks. This leads to the well-founded conclusion that a rock-mass stability monitoring system is necessary for the cave [27].

**Figure 1:**
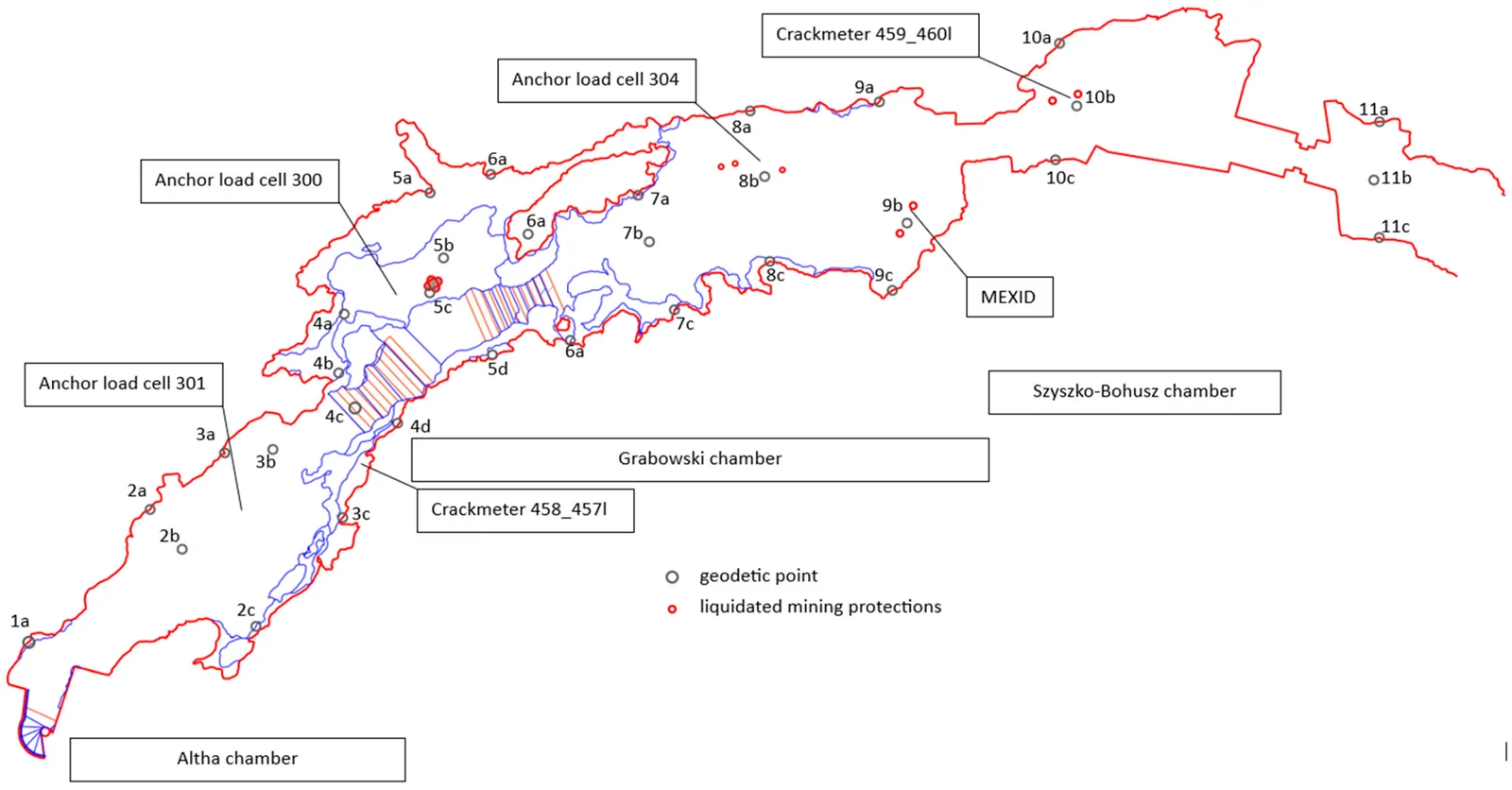
Schematic layout of the geotechnical monitoring system and survey points in the Dragon’s Den Cave. The red line indicates the maximum outline of the cave, the blue line the ground level.

Between April 2020 and April 2021, the cave underwent comprehensive renovation and reinforcement works. More than 1500 m of steel and glass epoxy rock bolts were installed in the surrounding rock mass, and of the ten timber cribs and wooden posts previously present, only a single reinforced column was retained. In view of the replacement of support systems and the decision to construct an exhibition space above the cave, it was determined that a continuous rock mass monitoring system with an early warning function for threshold exceedance was required. The sensors are positioned at locations where mine support systems have been modified (Figure 1). This system is complemented by deformation measurements carried out at six-month intervals. The measuring sensors are either protected against mechanical damage or installed in locations inaccessible to tourists. The sensors are maintenance-free under the specified operating conditions.

The sensors acquire data at four-hour intervals; if a value exceeding the alarm threshold—established by a geotechnical engineer based on a separate analysis (1 mm for roof separation measurements, 2 mm for crack gauges, and 30 kN for anchor rod force measurements)—is detected, the readings are repeated, and the system immediately notifies the administrator and technical inspectors about the alarm.

The rock mass monitoring system was divided into three main groups:

1. Roof separation measurement stations—3 stations;
2. Stations for measuring the distribution of forces acting in rock bolts, based on strain gauge measurement of bolt elongation—3 stations;
3. Cave convergence measurement stations, which also serve as benchmarks and polygonal traverse points—10 stations.

The Wieliczka Salt Mine is an underground mining complex established in 1241 and operated continuously until 1996. It consists of approximately 300 km of galleries and chambers distributed across nine mining levels, reaching a depth of 327 m. In recognition of its exceptional cultural and historical value, as well as its extraordinary achievements in the fields of mining engineering and art, the Wieliczka Salt Mine was inscribed on the UNESCO World Heritage List on 9 December 1978 [28]. At present, no mining extraction activities are conducted within the mine; only mining protection works and the liquidation of underground voids are carried out. The chapel is approximately 12 m high, 18 m wide, and 54 m long. It constitutes the culminating point of the tourist route, which is visited annually by nearly 2 million tourists. Due to the large dimensions of the underground void, its depth, and the presence of surrounding chambers, the chapel exhibits typical rheological processes characteristic of salt deposits. These include convergence, rotation of sidewalls, uneven floor subsidence, and horizontal displacements of control points [29].

**Figure 2:**
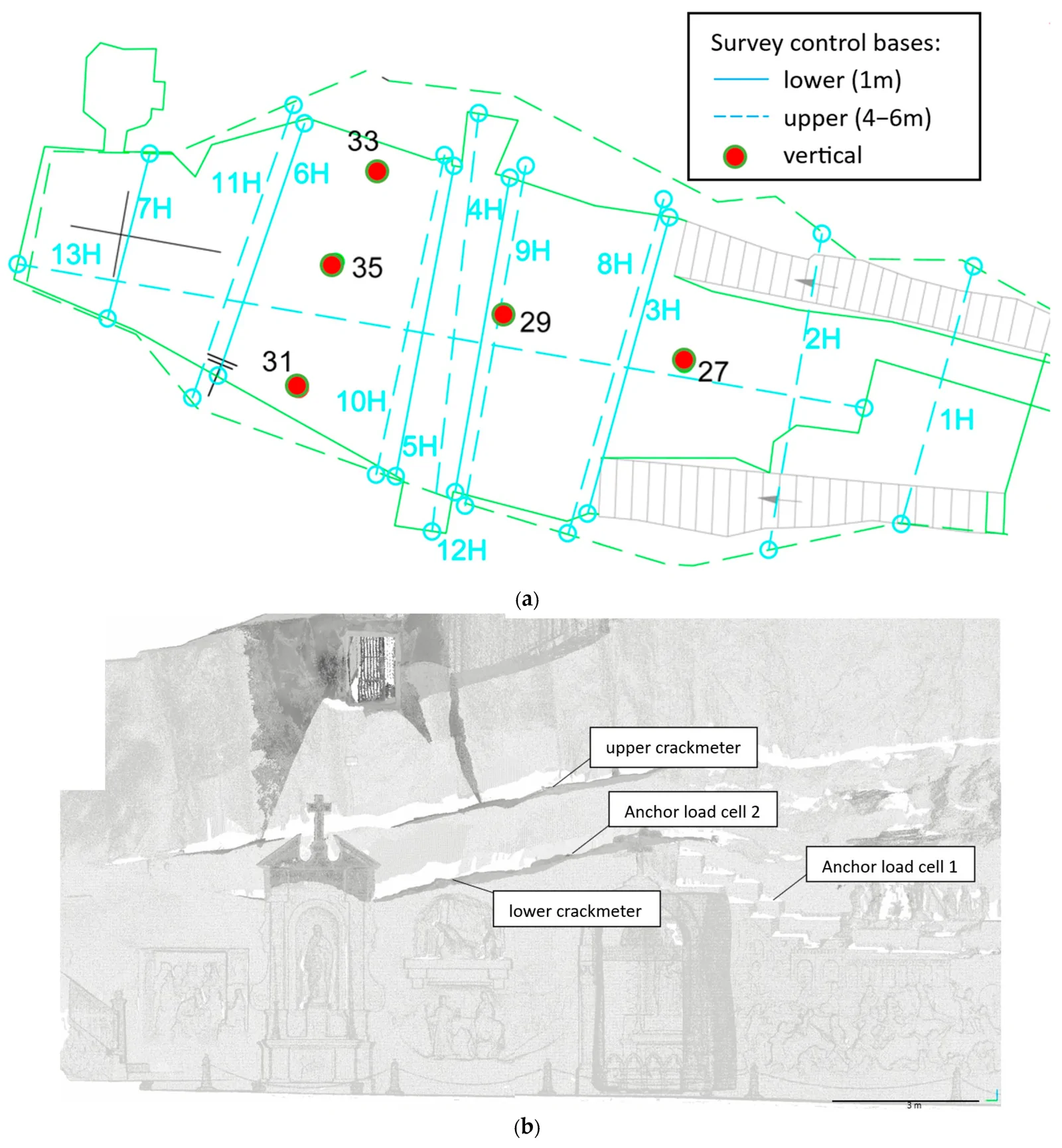
Location of (**a**) existing measurement bases and (**b**) installed sensors within the chapel.

In the area of the northern sidewall of St. Kinga’s Chapel, which underwent intensive and advanced rock bolting between 2021 and 2022, four measurement stations (six sensors in total) were installed in December 2023 as part of a salt rock mass deformation monitoring system. The system was designed as a fracture separation monitoring network operating in a quasi-continuous mode (Figure 2b). According to the schematic layout, two stations were installed to measure the forces acting in rock bolts, and two stations were dedicated to monitoring fracture separation. Each fracture monitoring station consists of two transversely oriented crack meters (deformation gauges) based on vibrating wire technology.

The measuring sensors are installed in locations inaccessible to tourists. These sensors complement a long-standing monitoring system already present in the chapel, which consists of a set of roof separation sensors (designated as stations 27, 29, 31, 33, and 35) and a network of points used for convergence measurements (Figure 2a). In the immediate vicinity of the installed sensors, base reference points 4H and 5H are located (marked with solid lines as floor-level convergence bases), as well as points 9H, 12H, and 10H (marked with dashed lines, positioned on a ledge approximately 5 m above the floor).

The sensors acquire data at 12-h intervals; if a value exceeding the alarm threshold—established by a geotechnical engineer based on a separate analysis (2 mm for roof delamination measurements, 2 mm for crack sensors, and 50 kN for anchor rod force measurements)—is detected, the measurement is repeated, and the system immediately notifies the administrator about the alarm.

Due to the specific nature of the measurement sites—namely, a natural karst cave and a salt mine, both inscribed on the UNESCO World Heritage List—very stringent requirements had to be adopted for the installation of monitoring sensors. First, sensor locations had to ensure their protection by preventing access by unauthorized persons, while simultaneously enabling continuous monitoring of the selected measurement points. Installation works were required to be carried out in close cooperation with heritage conservation authorities. This included, among others, prohibition of percussive drilling within the chapel, concealment of cables within natural fractures or drilled boreholes, and the avoidance of any visual exposure of sensors. Secondly, high humidity conditions in the karst cave and the aggressive saline environment in the salt mine necessitated maximum durability of sensor housings and a high degree of system tightness, ensuring long-term reliability of both mechanical and electronic components. Radio communication posed an additional challenge due to signal reflections and shielding caused by complex rock geometry, which ultimately required the implementation of wired data transmission. For the realization of the monitoring system, sensors manufactured by SISGEO, Milan, Italy were selected.

To supplement geological knowledge, personnel drilling boreholes for rock bolting and other purposes are required to prepare detailed borehole logs. These logs provide additional information on rock quality and continuity behind the exposed excavation surface, as further into the rock mass there may occur voids, weaker clayey rock fractions (in the cave), or salt of reduced hardness. This information also enables the development of a three-dimensional model of the rock mass surrounding the underground void, which may indicate the need to enhance the applied support measures by, for example, installing longer rock bolts or additional timber cribs.

**Figure 3:**
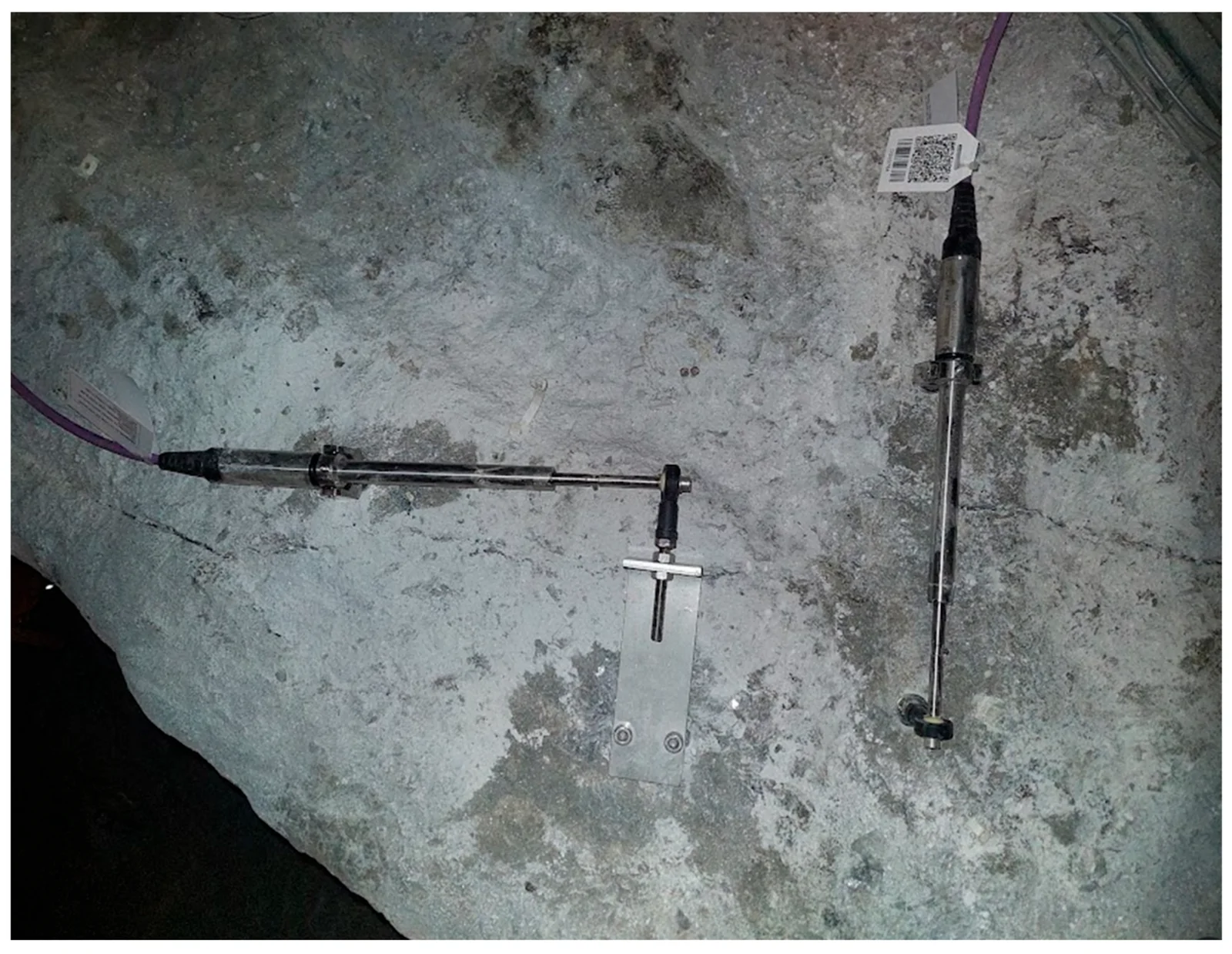
D313F050VW electronic crack meter operating in two axes in St. Kinga’s Chapel.

Observations of fractures and separations in planes perpendicular to the exposed excavation surface were carried out through the installation of electronic crack meters (deformometers), enabling measurements in two planes. Vibrating wire sensors with a measurement range of 50 mm were applied (Figure 3), equipped with an integrated temperature sensor allowing for the reduction of temperature-induced effects on sensor readings. The sensors are used to monitor relative displacements between two opposing surfaces with a resolution of 0.01 mm and overall accuracy less than ±0.3% FS [30]. The electrical transducer is enclosed within a stainless-steel housing mounted on a base plate, ensuring mechanical stability and durability under adverse environmental conditions.

**Figure 4:**
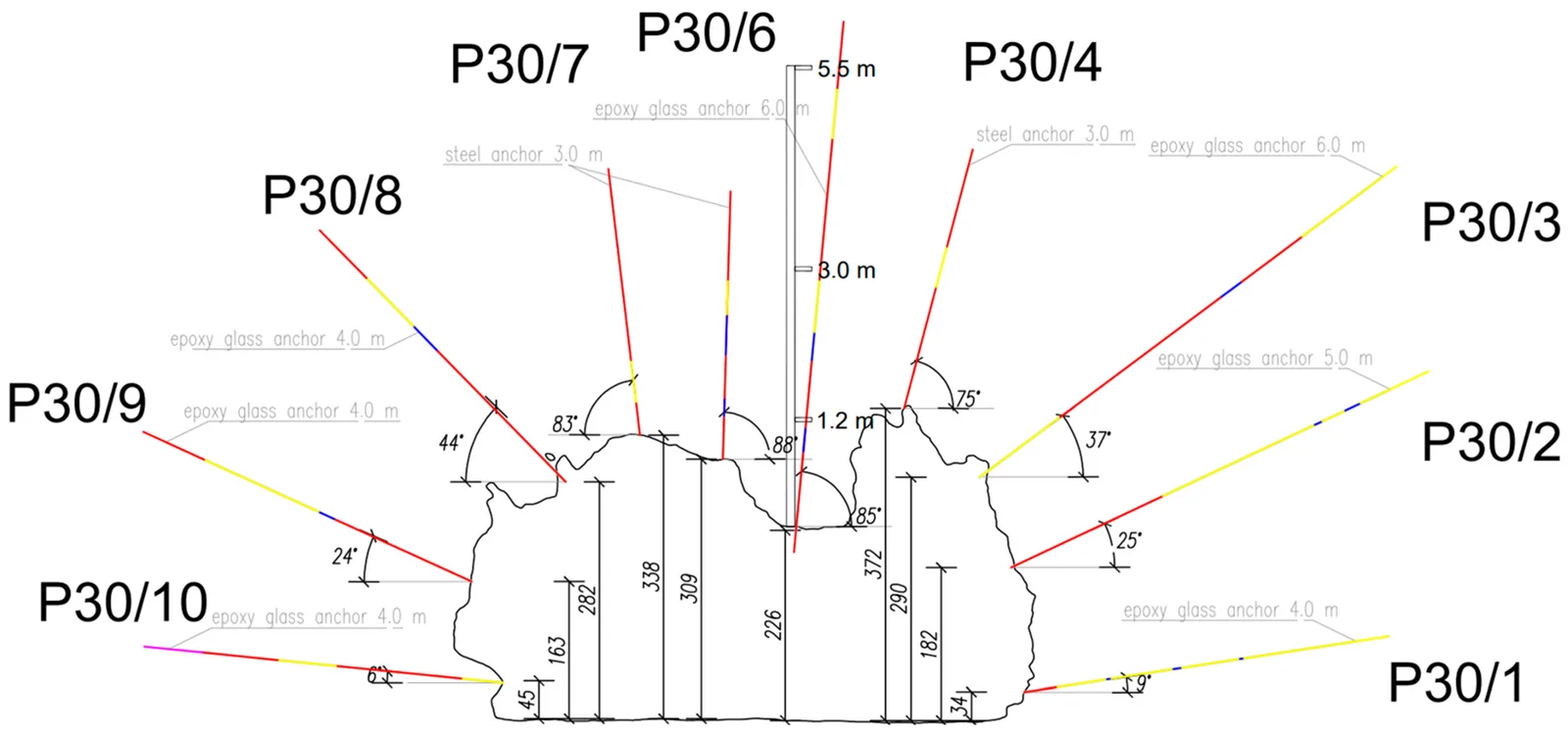
Location of the MEXID test anchor above the rock overhang, with borehole data marked.

In the Dragon’s Den Cave, within the largest chamber, a multipoint extensometer installed in a borehole was applied to monitor significant roof separations identified within the rock mass. The device performs measurements of incremental length changes and is of the MEXID type (Figure 4). Taking into account the heritage classification of the monitored object, a miniaturized sensor was selected, requiring a borehole with a diameter of only 50 mm and allowing the entire device to be fully concealed within the rock mass. Externally, only the power and data transmission cable is visible. The location was determined by the planned removal of a pillar supporting a large rock overhang; however, drilling revealed a lack of rock continuity (in Figure 4, intact limestone is marked in red, voids in blue, and rock rubble mixed with clay in yellow—pre-anchoring boreholes were drilled at 1-m intervals along similar cross-sections, enabling the spatial mapping of rock mass discontinuities above the roof and behind the sidewalls). The measurement range (with a resolution of 0.001 mm and overall accuracy less than ±0.025% FS [30]) and the lengths of individual measuring sections were defined as follows: 1.2 m (fracture at 1.0 m), 3.0 m (fracture at 2.8 m), and 5.5 m (fracture at 5.2 m).

Monitoring the load acting in rock bolts intended to replace a previous support system for a given location or a large roof area is a standard practice in underground engineering [31]. Bolt inspections are usually accompanied by pull-out resistance tests; however, in the present case, a different approach was adopted. Selected individual bolts were used, bonded only at their distal ends, while the bearing plate serves to assess the performance of the entire rock-bolting support system in the surrounding area.

**Figure 5:**
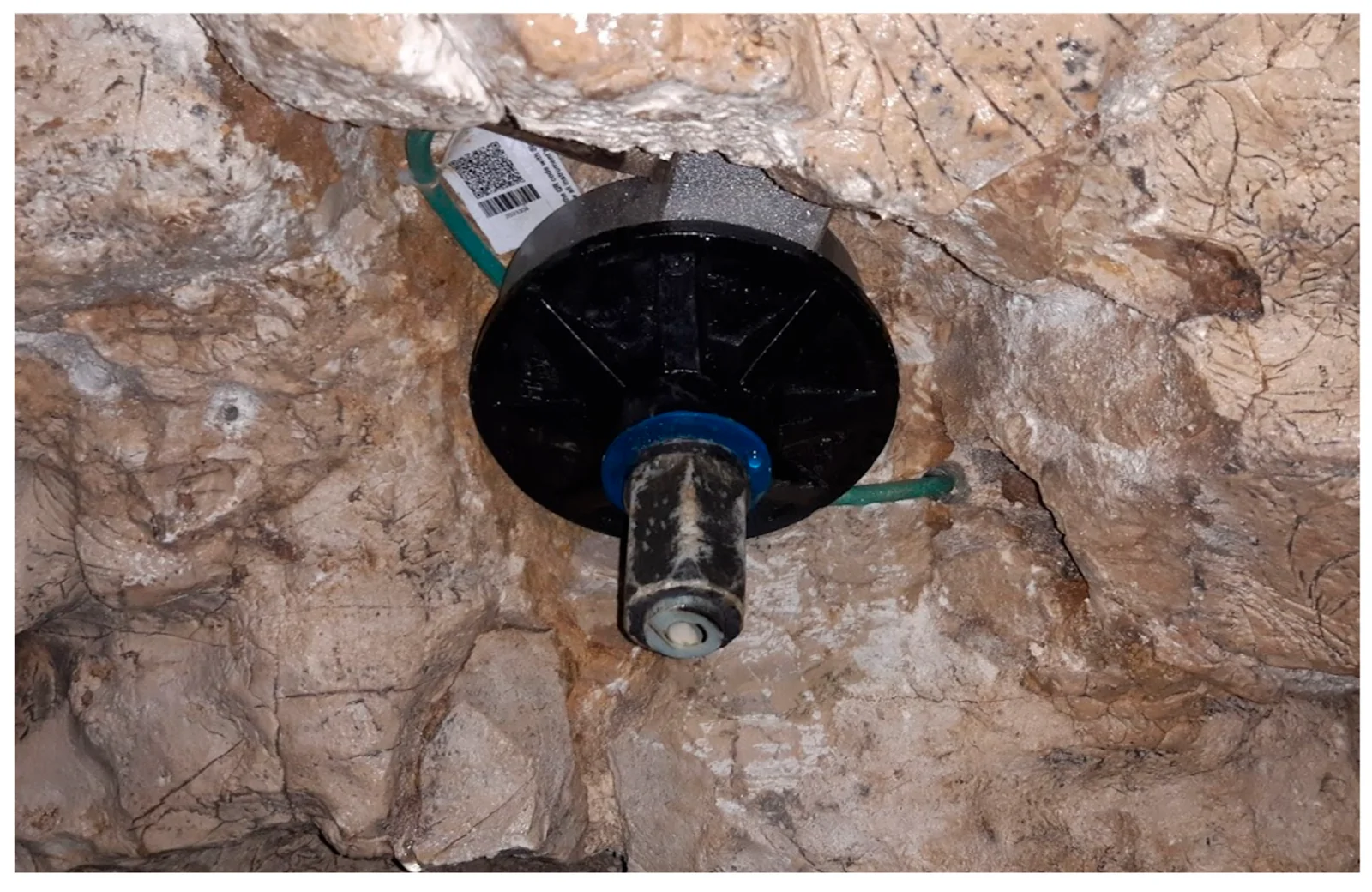
0L205V05000 electronic rock bolt load sensor installed in the Dragon’s Den Cave (measurement ring).

The electrical resistance rock-bolt load cell (Figure 5) consists of a ring-shaped stainless-steel housing in which three strain gauges are installed in a full Wheatstone bridge configuration (standard resistance of 1400 Ω). This sensor design minimizes the influence of uneven load distribution. The measurement range of the sensor extends from 0 to 500 kN, with an overload capacity of up to 150%. Data are recorded with a sensitivity of 0.001 mV and an accuracy of ±0.5% of full scale (FS) [30].

**Figure 6:**
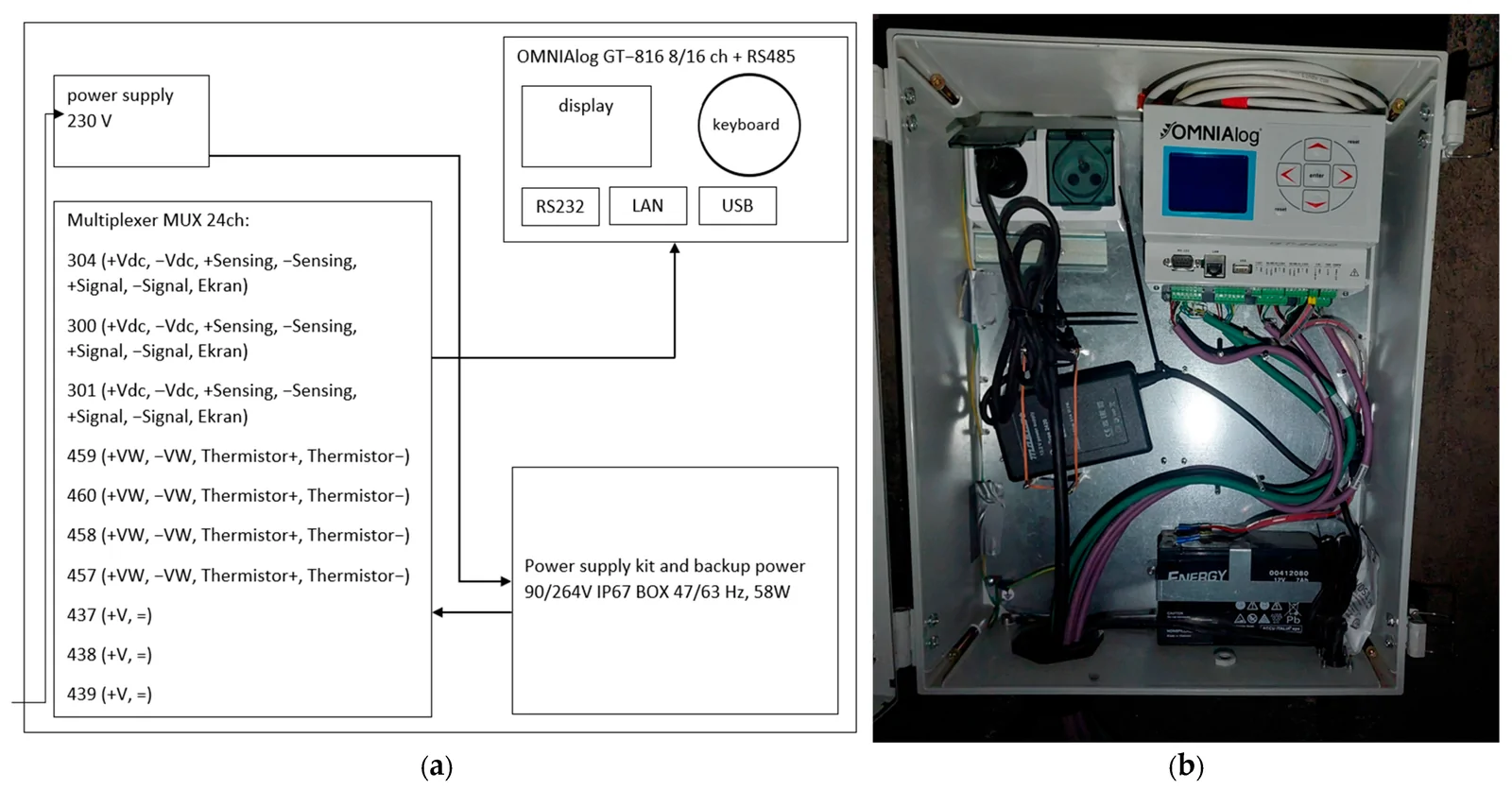
(**a**) Wiring diagram of the OMNIAlog enclosure; (**b**) body of OMNIAlog data logger.

The monitoring network described above is powered by an OMNIAlog data logger housed in a secure location (Figure 6b), while the recorded data are transmitted via a telecommunication network to the server room. In its current configuration (Figure 6a), the OMNIAlog unit transmits signals via a multi-core cable from the alarm control panel to the alarm signaling and visualization console, thereby providing an early warning system for threshold exceedance and alarm states. The system can also be expanded to include a router, enabling remote data transmission and off-site access to monitoring data.

**Figure 7:**
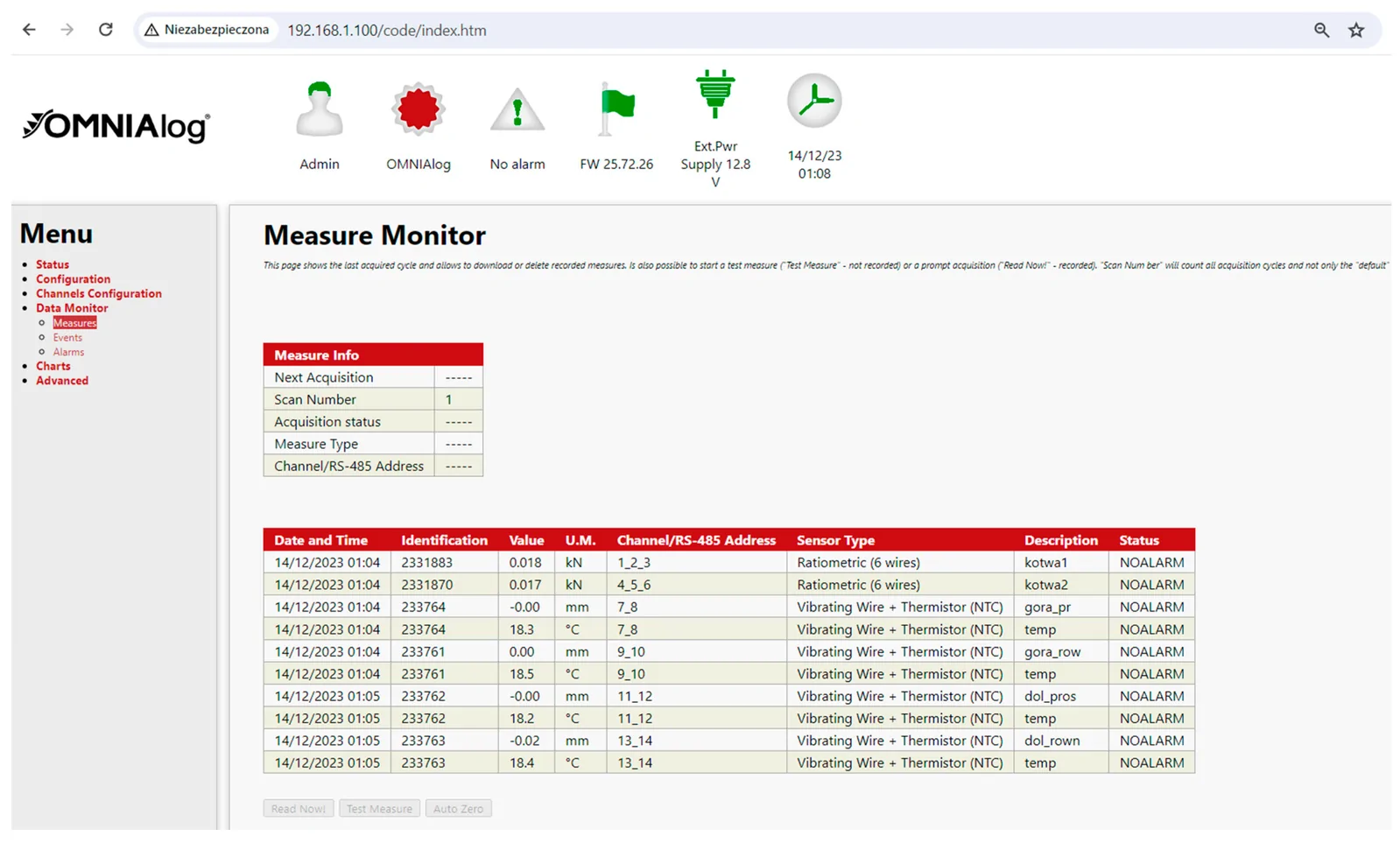
Console of OMNIAlog with last acquisition and system status.

Access to the console via a network connection is provided through a LAN port located in the OMNIAlog housing. Users gain access to the OMNIAlog console via an IP address using any standard web browser (Figure 7). Within the console environment, it is possible to configure sensors, enter calibration data, define alarm thresholds and alarm notification methods, import measurement data and alarm information, and generate graphical data visualizations.

The calibration data for the sensors were obtained from the certified SISGEO laboratory (Table 1), which performed the calibration using the direct comparison method and calibrated measuring instruments. To independently verify the results obtained, the calibration procedure was repeated after transportation in a university laboratory, using analogous measurement assumptions and comparing the sensor readings with the reference values.

Under field conditions, unlike the stable laboratory environment, an increased scatter of the results and additional changes in the readings were expected, mainly due to temperature, humidity, vibrations, and installation conditions. However, these changes should not lead to a significant modification of the metrological characteristics of the sensors, provided that the measurement system was properly installed and stabilized. The results obtained in the cave should therefore be treated as a verification of sensor performance under real-world conditions rather than as a direct equivalent of a laboratory reference calibration. The integrity of the measurements was assessed by analyzing the stability of the readings, repeatability, and agreement between the sensor characteristics and the results obtained during laboratory calibration. The analysis was performed for 12 measurement points of the 50 mm CRACKMETER sensor, covering an increasing cycle from 0 to 50 mm and a decreasing cycle from 50 to 0 mm. Table 2 compares the laboratory and field results obtained in the cave.

**Figure 8:**
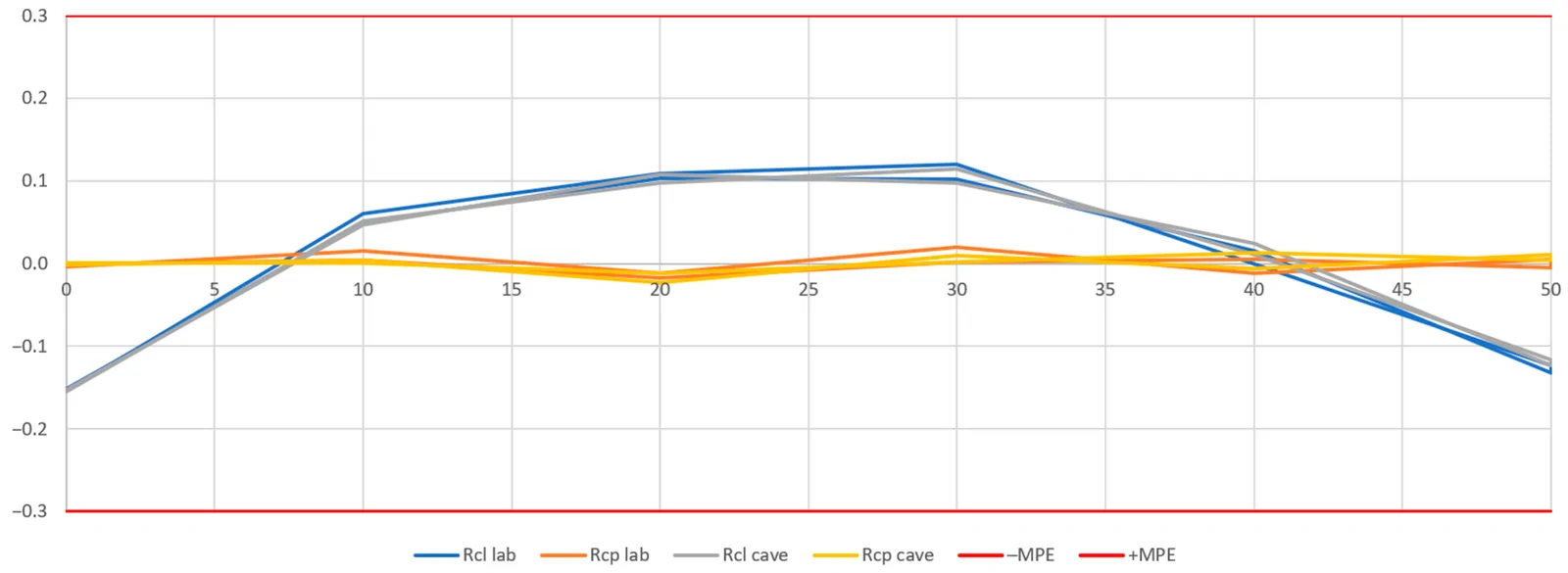
Chart of linear and polynomial error + U [mm] and Maximum Permissible Error (±MPE).

Table 1 and Table 2 include linear factors determined using the least-squares method and polynomial factors calculated by polynomial regression (1). It also presents values obtained using linear (3) and polynomial (5) corrections, as well as reference readings (6) and instrument readings (7). The reported errors represent the residual calibration error (2), including measurement uncertainty (U); they are calculated over the full measurement range and result from the combined effects of nonlinearity and hysteresis. The errors shown in the charts correspond to the sum of the error at each measurement point and the associated measurement uncertainty (Figure 8).

Statistical analysis of the results obtained for sensor no. 459 (Table 1 and Table 2), with a measuring range of 50 mm, showed that the polynomial characteristic, Rcp, provided a considerably more accurate representation of displacement than the linear characteristic, Rcl. The maximum error of the Rcp characteristic was approximately 0.0195 mm under laboratory conditions and 0.0233 mm during measurements in the cave, whereas for the Rcl characteristic, it reached approximately 0.15 mm. The mean error values were close to zero, indicating no significant systematic error. The maximum hysteresis did not exceed approximately 0.020 mm, and the differences between the laboratory and field results were small. The results indicate that the change in operating conditions did not cause any significant deterioration in the sensor’s measurement characteristics.

Analogous tests were conducted for all sensors, yielding consistent results. Therefore, the calibration parameters provided by the manufacturer were entered for all sensors, using the polynomial factors.

## 3. Results

Data stability depends primarily on the quality of sensor installation and the routing of cable paths. The installation of crack meters is based on mounting plates (visible in Figure 3), which are drilled and fixed using expansion anchors for concrete and rock or bonded with rigid chemical anchors. Other sensors require wider and deeper boreholes—reaching up to several tens of meters—followed by stabilization of the anchors using specialized adhesives selected according to local geological and environmental conditions. Although each conductor within the cable, as well as the cables themselves within their rubber sheath, is shielded, the manufacturer recommends routing them inside separate conduits or cable trays. Additionally, cable routes should not intersect electrical power cables; where this cannot be avoided, supplementary shielding should be applied. The author’s experience indicates that alternating-current power cables with voltages up to 230 V do not significantly affect the stability of sensor readings; however, for safety reasons, all crossing points were additionally shielded.

**Figure 9:**
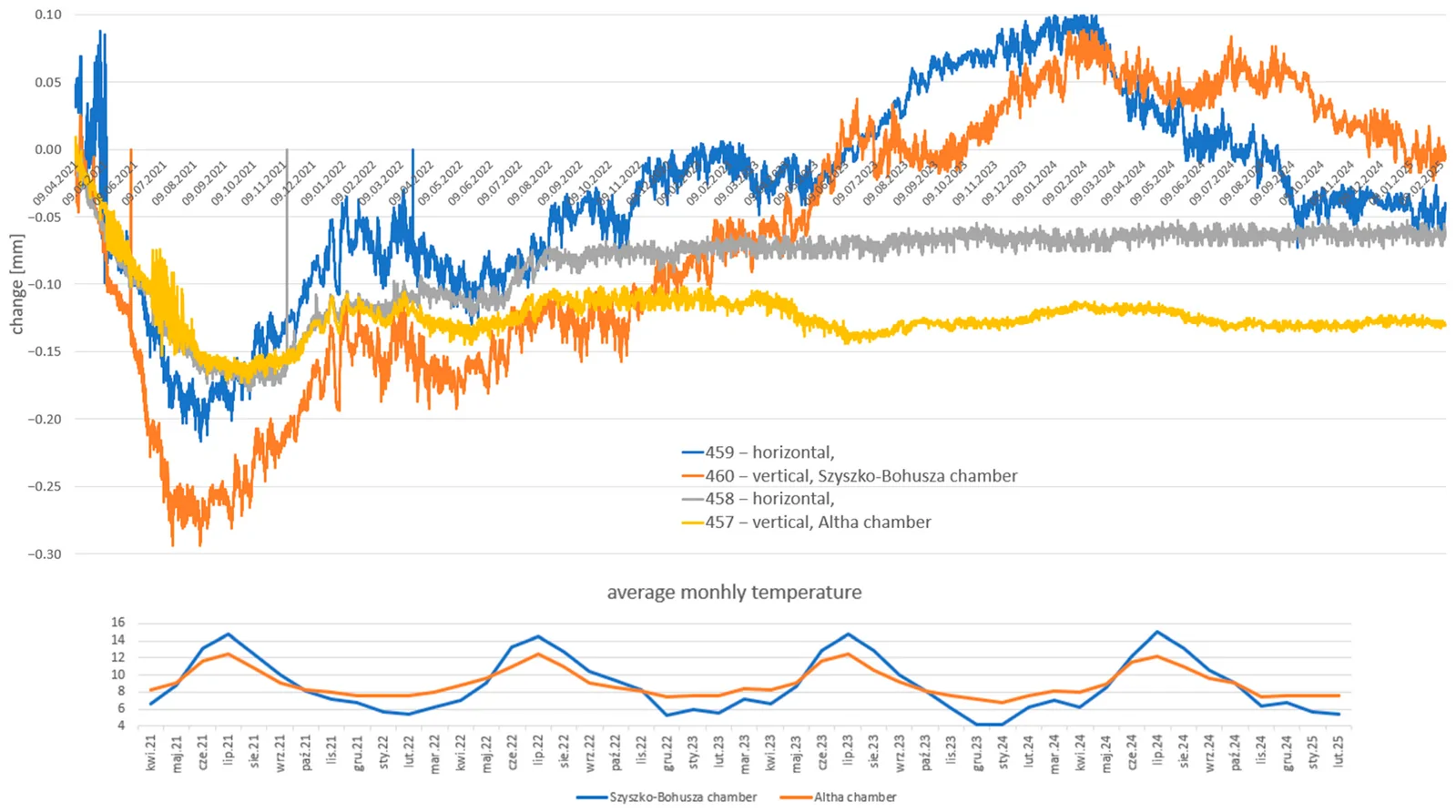
Changes recorded by electrical wire crack meters in the Szyszko–Bohusz and Altha chambers compared with the average monthly temperature in Dragon’s Den Cave.

Overall, the data stability for all sensors can be assessed as very high. During four years of observations in the Dragon’s Den Cave and two years in St. Kinga’s Chapel, the monitoring system triggered an alarm on only two occasions. In both cases, the alarm was caused by the reception of a non-numerical value (NaN: *Not a Number*), which was most likely the result of a voltage surge or an internal system error. A third anomaly visible in the time series (Figure 9) was deliberately induced as part of a test intended to demonstrate the correct operation of the system. In order to eliminate false alarms, the system was configured so that upon detection of an alarm condition, the measurement is immediately repeated three times. An alarm is triggered only if all three results are consistent.

Data from the Dragon’s Den Cave have been collected since April 2021. The observed changes over the entire monitoring period are very small and, after four years, have essentially returned to the initial baseline level. Neither the rate of change (expressed in mm/year) nor the total displacements recorded during the observation period have ever exceeded the thresholds defined by the authors as significant.

The first graph presents data obtained from crack meters installed in the Szyszko-Bohusz and Altha chambers. After an initial system stabilization phase—characteristic of telemetric sensors—a period of stable operation followed, with only minor variations observed (Figure 9). These variations are interpreted as being primarily driven by thermal effects rather than by actual changes in the geometry of the structure. Below, the average monthly temperatures recorded for each chamber are presented.

**Figure 10:**
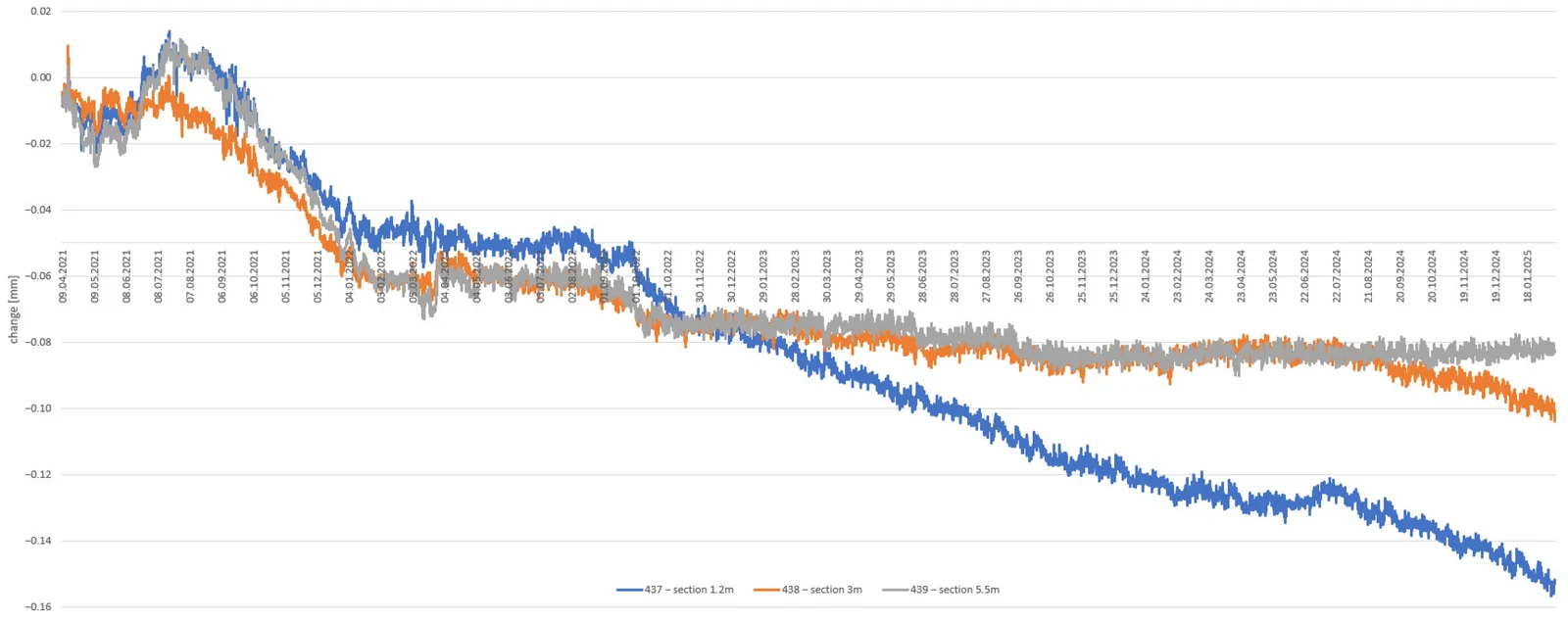
Changes in sensor readings recorded by MEXID downhole anchors [mm].

The second type of sensor measuring metric values described in this study is the MEXID system, used to assess roof separation in the area where former supports were removed and replaced with a rock-bolting support system. The shortest measuring section, monitoring the shallowest fracture, exhibits changes occurring at approximately twice the rate observed in the deeper sections. Nevertheless, the total recorded values remain very small, reaching up to 0.16 mm, which corresponds to an average rate of approximately 0.04 mm/year (Figure 10).

**Figure 11:**
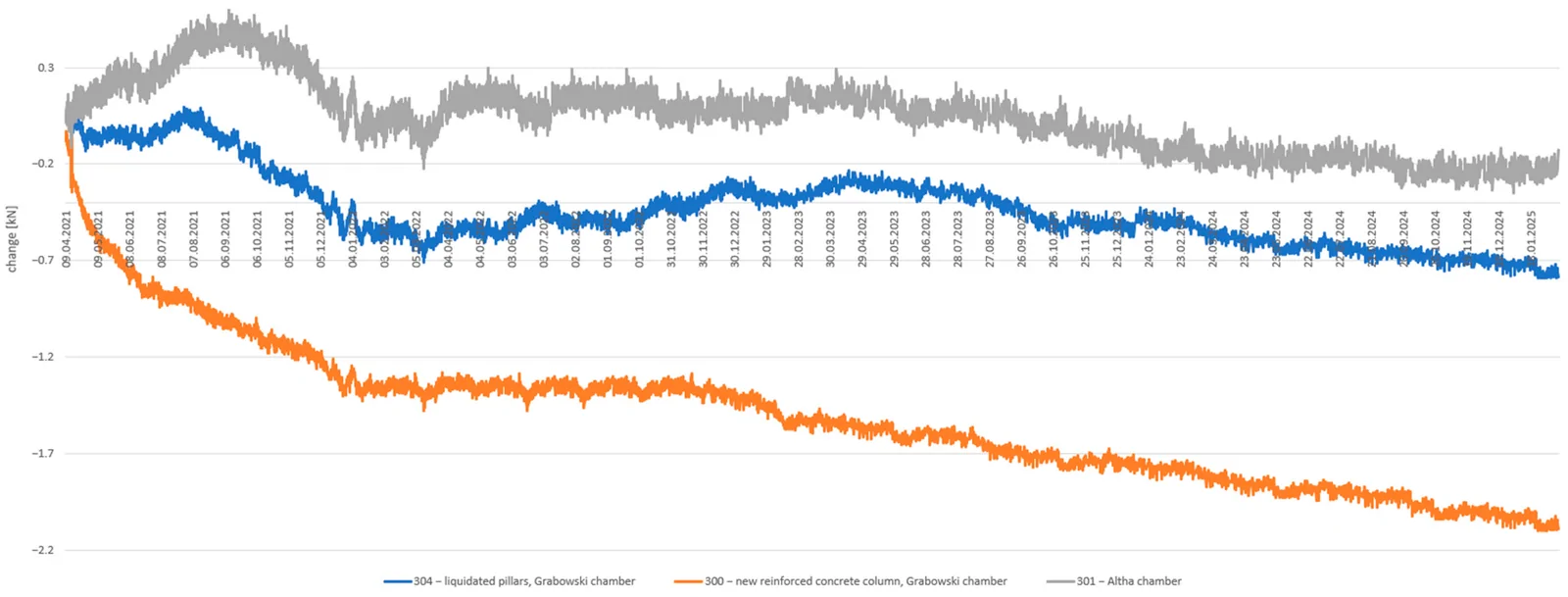
Changes in readings of the rock bolt load sensors [kN].

The final type of sensor installed in the Dragon’s Den Cave comprises rock bolt load sensors, which demonstrate the stability of the support system and confirm the validity of replacing previous support solutions with a dense rock-bolting system (Figure 11).

**Figure 12:**
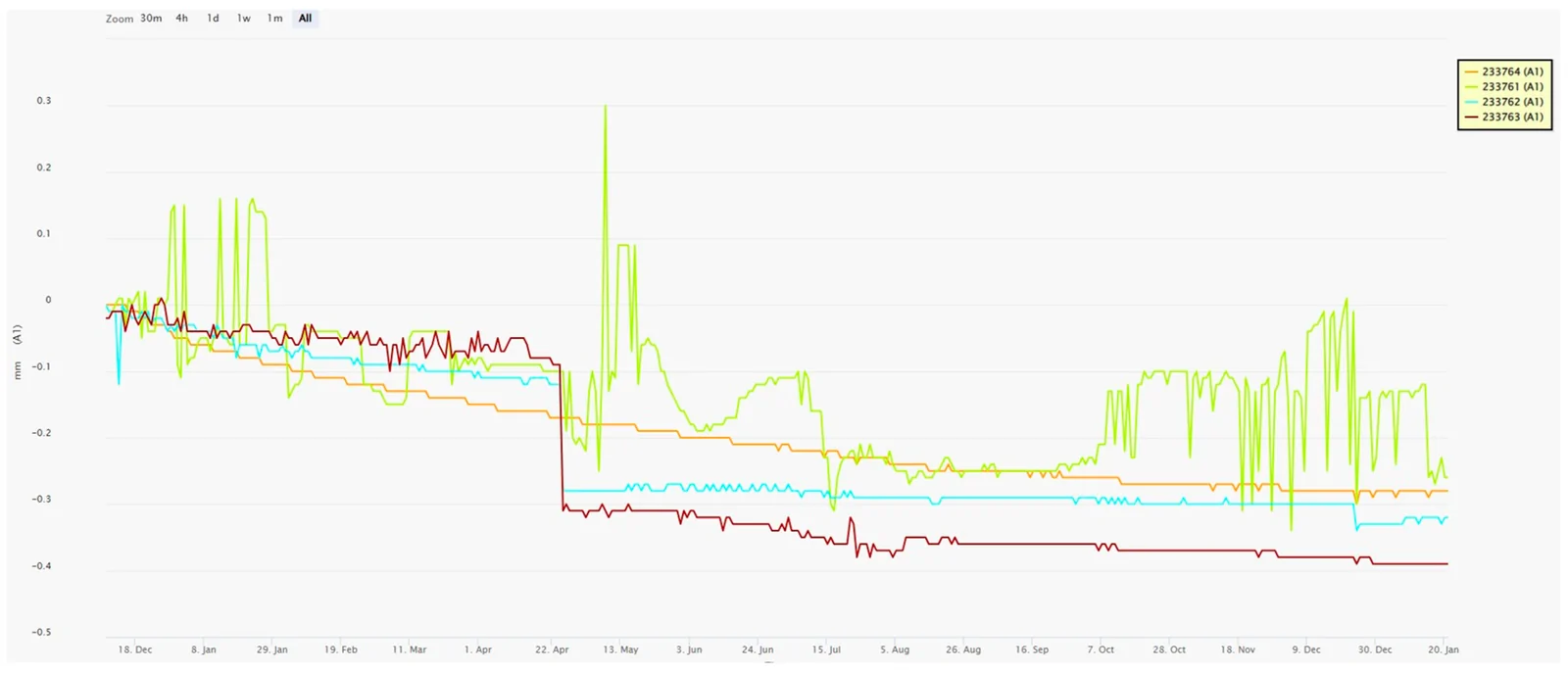
Readings of vibrating wire crack meters in St. Kinga’s Chapel.

In St. Kinga’s Chapel, the sensors also exhibit relative stability of readings, although their operation is occasionally disturbed by cleaning activities involving the removal of dust from rock ledges (Figure 12).

### 3.1. Determination and Verification of Temperature Coefficients

One of the fundamental challenges in structural monitoring is temperature variation and the associated thermal expansion of both the monitored structures and the sensors themselves. For measurement readings, direct exposure to sunlight is particularly detrimental [32]. In the case of the analyzed underground objects, solar radiation is irrelevant; however, based on experience gained from surface structures, the authors decided to investigate the influence of natural temperature variations.

In the Wieliczka Salt Mine, temperature and humidity are closely monitored and actively regulated through an extensive ventilation and air-conditioning system to preserve the salt sculptures. In contrast, in the Dragon’s Den Cave, temperature is not actively controlled, while humidity regulation is achieved only by opening windows in the towers constructed above the cave. These openings function as ventilation shafts (exhaust chimneys) enabling air migration. As a result, particularly in the area near the cave entrance—in the Szyszko-Bohusz Chamber—annual temperature amplitudes reach approximately 10 °C, with daily fluctuations of up to 5 °C.

For their analysis, the authors selected extensometers in the Dragon’s Den Cave that are most susceptible to temperature fluctuations and originally equipped with a thermometer. The other sensor types were installed within the rock mass and are therefore exposed to much smaller temperature variations. The analysis of the effect of temperature on extensometer readings was carried out in two stages. In the first stage, regression coefficients were determined using a representative, synchronized dataset with four-hour resolution. In the second stage, it was verified whether these coefficients remained applicable after being applied to monthly averages covering the entire available observation period.

This distinction is important because the two stages addressed different statistical questions:

(a) The four-hour analysis was used to determine the short-term relationship between temperature and measured displacement and identify any response delay [December 2021–March 2023].

(b) The monthly analysis for whole dataset [April 2021–February 2025] was used to assess whether the relationship remained useful over the multi-year series, including changes in mechanical and measurement conditions.

#### 3.1.1. Regression Model for Four-Hour Data

For each extensometer, the following multiple linear regression model was applied [33]:

D(t) = β_0_ + βT T(t − τ) + βtime t + ε(t),

where:

D(t) is the extensometer reading;

T(t − τ) is the temperature with a time delay τ;

βT is the temperature sensitivity coefficient;

βtime describes the long-term measurement trend;

t represents time;

ε(t) is the residual component of the model;

τ is the delay between a temperature change and the extensometer response.

Delays ranging from 0 to 48 h were tested at four-hour intervals. For each variant, βT; its 95% confidence interval, *p*-value, R^2^, adjusted R^2^, and RMSE; and the AIC and BIC criteria were calculated (Table 3). The best fit for all four extensometers was obtained for a delay of τ = 0 h. As the delay increased, the quality of the fit systematically deteriorated. For example:

- For extensometer 457, R^2^ decreased from 0.940 at τ = 0 h to 0.529 at τ = 48 h;
- For extensometer 458, it decreased from 0.790 to 0.542;
- For extensometer 459, it decreased from 0.981 to 0.399;
- For extensometer 460, it decreased from 0.956 to 0.406.

At the same time, RMSE increased with the delay. Therefore, the model with τ = 0 h was selected as the reference model.

All βT coefficients were statistically significant for the four-hour data, and their 95% confidence intervals did not include zero. The coefficients were negative. With the adopted sign convention, this means that an increase in temperature was accompanied by an average decrease in the extensometer reading.

The four-hour models showed a good fit. The highest R^2^ values were obtained for sensor 459—0.9814—and sensor 460—0.9562. For sensor 457, R^2^ was 0.9397, while for sensor 458, it was 0.7902. This means that during the representative four-hour observation period, the model incorporating temperature and a time trend explained approximately 79% to 98% of the variability in the measurements.

These results supported the use of the obtained βT coefficients as empirical parameters representative of the analyzed measurement system and the selected observation period. However, they did not demonstrate that the coefficients remained stable throughout the entire multi-year series.

#### 3.1.2. Application of the Coefficients to Monthly Averages

In the second stage, the βT coefficients determined from the four-hour data were applied to monthly average extensometer and temperature measurements covering the period from April 2021 to February 2025. In this stage, the βT coefficients were not re-estimated using the monthly data. The purpose was to verify whether the relationship identified from the high-resolution data could also describe the long-term changes observed after aggregating the data to a monthly scale (Table 4).

In the monthly models, the βT coefficient was not statistically significant for any of the four sensors at the significance level α = 0.05. In all cases, the 95% confidence intervals for the monthly βT coefficients included zero. Therefore, after converting the data to monthly averages and accounting for the time trend, there were no statistical grounds to reject the null hypothesis:

H_0_: βT = 0.

This result does not mean that temperature had no physical effect on the measurement system. It means that after transitioning to monthly averages and considering the entire observation period, the temperature relationship identified from the four-hour data was not sufficiently stable or strong for its effect to be statistically distinguished from zero.

Sensor 459 was closest to the significance threshold, with *p* = 0.098; however, this value also did not meet the α = 0.05 criterion. Therefore, there are no grounds for considering the monthly βT coefficient statistically significant for the entire period.

The quality of the monthly models was clearly lower than that of the four-hour models. The models explained approximately 7% to 74% of the variability in the monthly measurements. The fit was very weak for sensor 457. For sensors 458 and 459, the models explained approximately 40–43% of the variability, while the highest R^2^ value was obtained for sensor 460. However, the R^2^ values should be interpreted together with the significance tests. The high R^2^ value for sensor 460 does not indicate a significant temperature effect because the *p*-value for βT was 0.568. The high R^2^ was primarily associated with the presence of a strong and statistically significant time trend. The time trends were approximately 0.0741 mm/year for sensor 460, 0.0360 mm/year for sensor 459, and 0.0182 mm/year for sensor 458. These values were substantially greater than the effect that could be reliably attributed to the monthly temperature variation.

This result demonstrates that a high R^2^ value cannot be equated with a good description of the temperature effect. A model may achieve a high R^2^ mainly because it reproduces a long-term measurement trend. This was particularly evident for sensor 460. The RMSE values for the monthly models ranged from 0.0209 mm for sensor 457 to 0.0516 mm for sensor 459. They were therefore substantially higher than the errors obtained for the four-hour data, for which RMSE ranged from 0.00153 to 0.00363 mm. This indicates a considerably greater uncertainty when describing long-term changes using a model based on a constant temperature effect and a linear time trend.

Overall, the results indicate that temperature exerted a clear short-term influence on the extensometer readings, but this relationship was not sufficiently stable to serve as a universal correction over the entire multi-year observation period.

### 3.2. Geodetic Measurements in Dragon’s Den

Geodetic measurements conducted in Dragon’s Den provide an independent spatial assessment of deformation within the cave. Unlike the extensometers, which monitor selected local zones—particularly areas where the rock support system has been modified—the geodetic points are distributed along measurement profiles throughout the cave. Consequently, the two monitoring systems observe different aspects of the rock mass response and should be regarded as complementary. The geodetic network consists of points located in several profiles (Figure 1). Their arrangement makes it possible to assess the spatial distribution of displacements and determine whether the changes observed locally by the extensometers are accompanied by a broader deformation of the cave or the surrounding rock mass.

**Figure 13:**
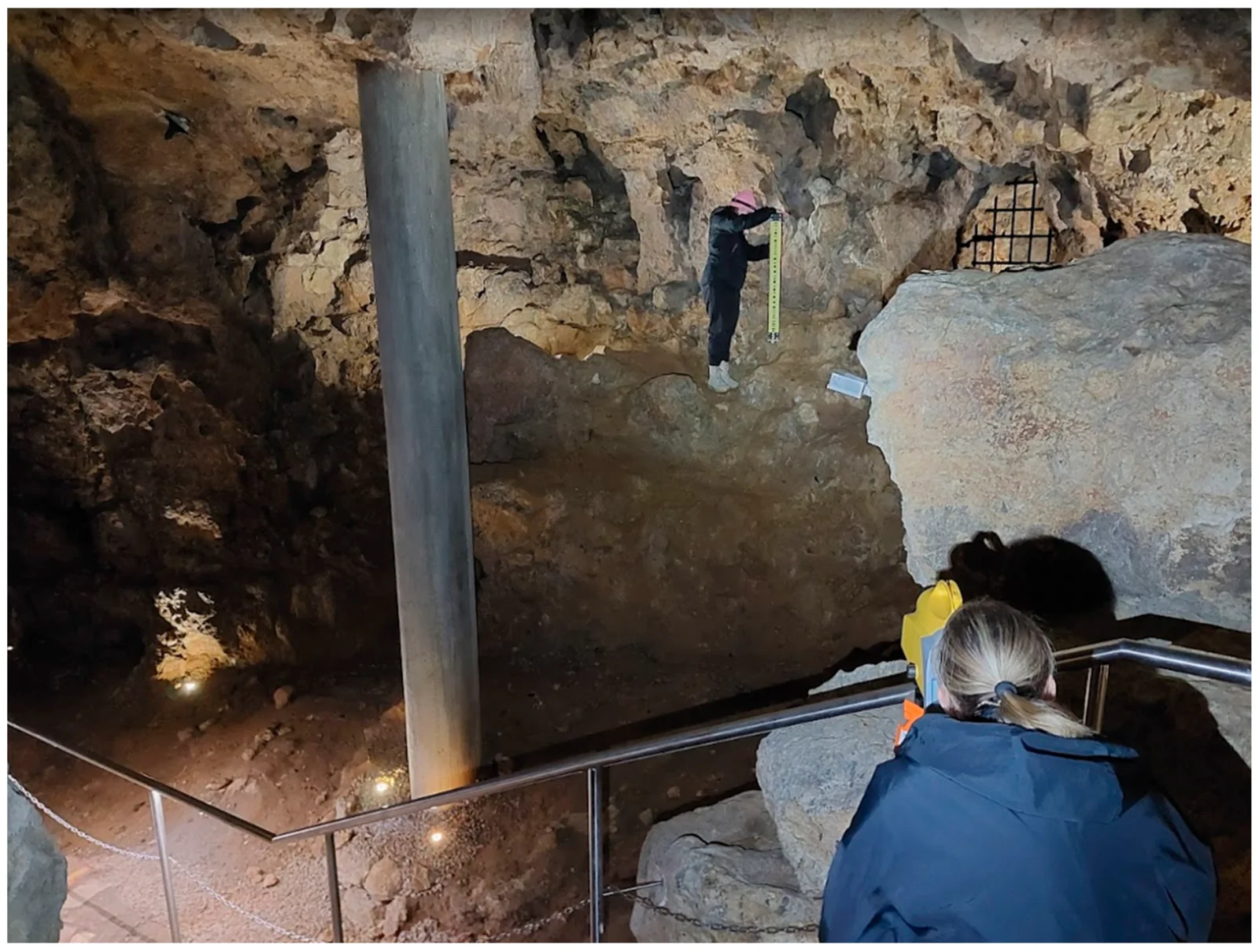
Precise leveling of convergence bases in Dragon’s Den Cave.

Figure 13 shows the process of precise leveling of distributed points constituting convergence bases for areas classified as particularly vulnerable. In this case, a new reinforced-concrete column replacing the former timber crib system has opened the space; however, the mass of karst rock consolidated with rock bolts and grouting that it supports is particularly susceptible to deformation. A rock bolt load sensor is visible in the upper left corner of the photograph.

The analysis presented here focuses primarily on a comparison between Series VII, conducted on 7 November 2024, and Series VIII, conducted on 13 February 2025 (all series have been taken every 6 months since March 2021). The latest measurements indicate that the cumulative displacements remained small. The maximum recorded horizontal displacement was approximately 2.6 mm, while the maximum vertical displacement was approximately 2.4 mm.

A comparison of Series VIII with Series VII does not indicate a systematic increase in deformation. At several points, including points 1A, 2A, 2C, 3A, 4A, and 5A, the cumulative displacement values were lower in Series VIII than in Series VII. At other points, small increases or practically unchanged values were observed. Thus, the changes did not show a consistent direction or a spatially coherent pattern that would indicate progressive destabilization of the entire rock mass.

A detailed comparison of selected points between the seventh and eighth measurement series is presented in Table 5. The table includes points for which the observed changes are representative of the overall pattern of the results.

As shown in Table 5, there was no uniform direction of change between Series VII and Series VIII. At some points, the displacement values remained practically unchanged, whereas at others, slight increases or decreases were observed. These changes are generally comparable to the measurement accuracy and therefore do not, on their own, provide sufficient grounds for concluding that progressive deformation is occurring.

The approximate measurement accuracy is ±1 mm for the horizontal component and ±0.5 mm for the vertical component. Consequently, differences of a similar magnitude should be interpreted with caution. They cannot be treated as evidence of actual progressive deformation unless they are confirmed by subsequent measurement series and form a consistent spatial and temporal pattern. Thus, the geodetic results provide no evidence of a coherent, progressive deformation of the cave at the investigated spatial and temporal scales, while confirming the need to interpret local sensor trends in their broader spatial context.

## 4. Discussion

The results obtained in the Dragon’s Den Cave and St. Kinga’s Chapel indicate stable behavior of the monitored locations during the investigated observation periods. The absence of significant displacement trends and structural alarm events should, however, be interpreted as evidence of no detectable progressive deformation at the instrumented locations and within the available time intervals, rather than as proof that all deformation processes in the surrounding rock mass are absent. Underground rock masses are heterogeneous, and deformation may develop locally along discontinuities, weak zones, or previously modified support systems.

The installation of an integrated monitoring system was justified by the specific conditions of both sites. The Dragon’s Den Cave underwent extensive reinforcement and modification of its former support system, whereas St. Kinga’s Chapel is affected by the long-term rheological behavior of salt rock. In both cases, the monitored structures are visited by large numbers of tourists and cannot be assessed solely through occasional visual inspections. The implemented system therefore provides an early-warning capability while limiting the need for frequent direct access to potentially hazardous or conservation-sensitive areas.

The results also demonstrate that local telemetric sensors and spatial geodetic measurements provide different, complementary types of information. Crack meters, load cells, and the MEXID extensometer are installed at selected locations considered particularly susceptible to crack opening, roof separation, or changes in support loading. They provide high-resolution observations of local behavior. In contrast, the geodetic network in the Dragon’s Den Cave consists of points distributed along measurement profiles independently of the locations of the telemetric sensors. It therefore provides a spatially broader assessment of the cave response.

This distinction is important when comparing the measurement results. The MEXID extensometer recorded a maximum change of approximately 0.16 mm at a selected roof-separation location, whereas the latest geodetic survey indicated cumulative displacements of approximately 2.6 mm horizontally and 2.4 mm vertically at the monitored survey points. These values are not directly interchangeable because they describe different quantities, measurement geometries, reference systems, and observation scales. The geodetic results did not show a coherent spatial or temporal pattern of progressive deformation. Differences between Series VII and VIII were generally comparable with the estimated measurement accuracy, approximately ±1 mm for the horizontal component and ±0.5 mm for the vertical component. Consequently, isolated changes between two consecutive survey series should not be interpreted as evidence of instability unless they are confirmed by subsequent measurements and form a consistent pattern.

The temperature analysis provides an additional example of the need for cautious interpretation. The four-hour data showed a statistically significant relationship between temperature and extensometer readings, with the best model obtained for a response delay of zero hours. The models explained between approximately 79% and 98% of the variability during the analyzed high-resolution period. However, when the coefficients derived from these data were applied to monthly averages covering the entire observation period, none of the temperature coefficients remained statistically significant. The relatively high coefficient of determination obtained for some monthly models was mainly associated with long-term trends rather than temperature.

This finding has two implications. First, temperature clearly affects the short-term response of the measurement system and should therefore be recorded together with structural measurements. Second, a temperature coefficient derived from a selected high-resolution period should be regarded as an empirical, period-specific correction rather than a universal physical parameter. Changes in ventilation, sensor conditions, support geometry, construction activities, or the statistical characteristics of the data may alter the observed relationship. Applying a fixed correction over several years could therefore remove genuine structural information or create an apparent trend.

The monitoring results also highlight the importance of distinguishing structural alarms from technical and environmental events. During the observation period, the system-generated alarms were associated with non-numerical values or system-related disturbances rather than confirmed structural changes. The use of repeated measurements before alarm confirmation is therefore an important element of the decision-support procedure. It reduces the risk that a temporary communication failure, voltage disturbance, invalid data packet, or isolated sensor reading will be interpreted as a structural hazard.

The reliability of the system depends not only on the sensors themselves but also on their installation and operating environment. Sensor protection, appropriate anchoring, cable routing, electrical shielding, data transmission, calibration, and periodic technical verification are essential for long-term operation. These requirements are particularly important in underground heritage sites, where high humidity, saline conditions, restricted access, complex geometry, and conservation constraints may affect both the measurement equipment and the maintenance procedures.

Terrestrial laser scanning complements the point-based and local measurements by documenting the geometry of larger surfaces. It is particularly valuable for identifying changes in the shape of chambers, roof surfaces, sidewalls, and support elements. However, its deformation-detection capability is generally lower than that of high-precision point measurements. The three techniques should therefore not be treated as competing methods. Their roles are different:

- Telemetric sensors provide continuous or quasi-continuous information at selected critical locations;
- Geodetic measurements provide periodic, spatially distributed control of the monitored structure;
- Terrestrial laser scanning provides comprehensive geometric documentation of surfaces and the overall underground space [34].

The practical value of this integrated approach is increased by the web-based monitoring dashboard. The dashboard should be understood not merely as a visualization interface but as part of a decision-support system for heritage conservation. It combines structural measurements, environmental observations, sensor status, alarm states, historical trends, and technical event logs. Separating structural alarms from technical notifications is particularly important because it prevents data transmission problems from being confused with physical instability. The simultaneous presentation of temperature and humidity data also allows users to assess whether a change in a structural signal may have an environmental or instrumental origin.

The proposed monitoring framework is therefore scalable and adaptable to other underground heritage sites. Its effectiveness does not depend on the use of a single sensor type or a single analytical model. Rather, it results from the integration of local high-resolution monitoring, independent spatial verification, environmental observations, robust data transmission, and expert interpretation. The present results support the continued operation of the system and the systematic repetition of geodetic and scanning surveys. Longer observation periods will be necessary to distinguish very slow deformation processes from measurement variability and confirm whether any local trend develops into a spatially coherent change.

**Figure 14:**
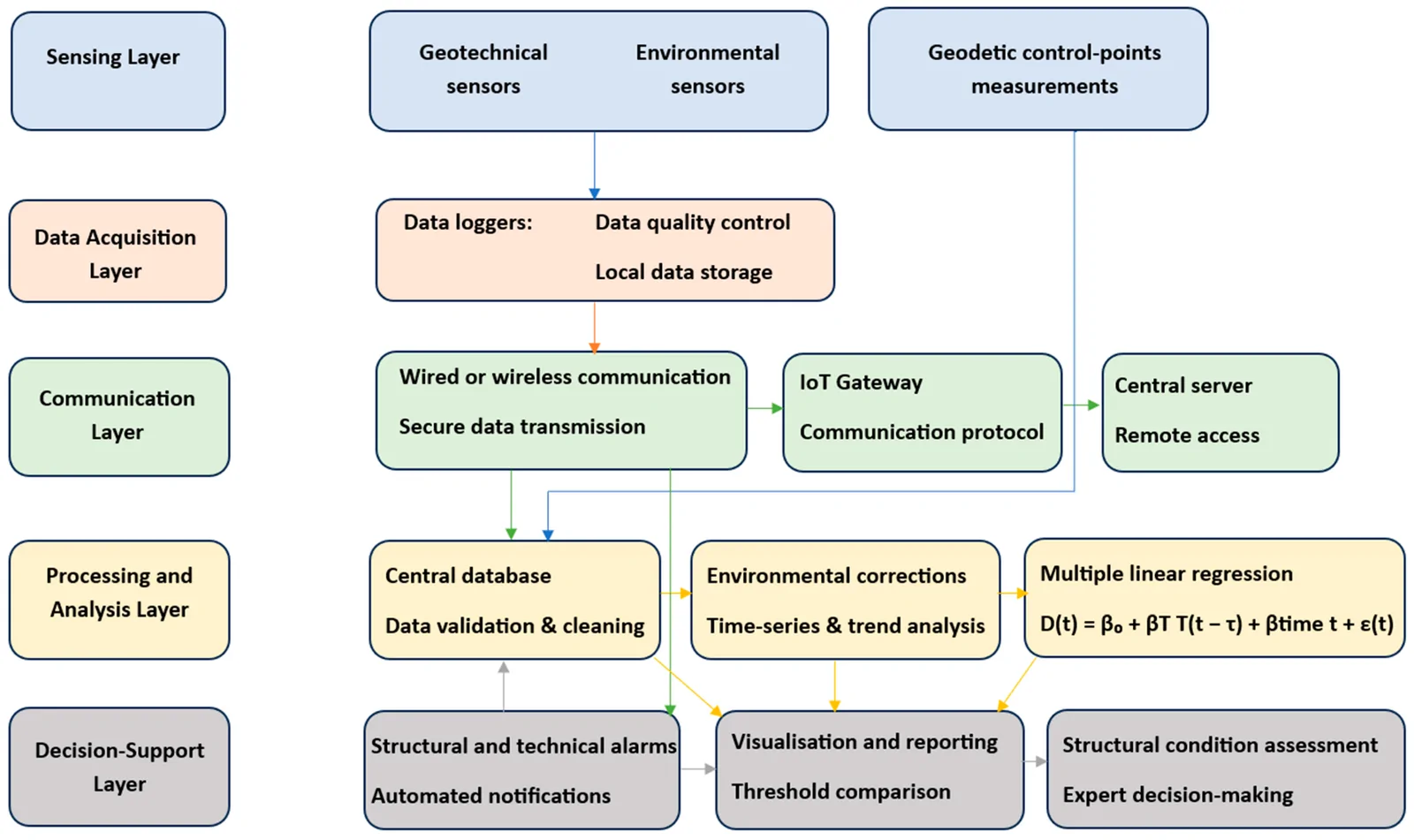
Functional architecture of the proposed IoT-based multi-sensor monitoring and decision-support system.

The results presented in this study demonstrate that reliable structural assessment requires the integration of measurements from different sensor types with environmental corrections and time-series analysis. In particular, the time-lagged multiple linear regression model can be incorporated into a broader monitoring and decision-support framework. Therefore, an IoT-based architecture is proposed to integrate sensing, data acquisition, secure communication, data processing, and expert interpretation, as illustrated in Figure 14.

## 5. Conclusions

This study demonstrated the feasibility of integrating quasi-continuous telemetric monitoring with periodic geodetic surveys and terrestrial laser scanning in underground heritage sites operating under demanding environmental and conservation conditions.

During the observation periods, the monitored locations in the Dragon’s Den Cave and St. Kinga’s Chapel exhibited stable behavior. The maximum displacement recorded by the MEXID extensometer in the Dragon’s Den Cave was approximately 0.16 mm, while the geodetic surveys indicated cumulative displacements of approximately 2.6 mm in the horizontal component and 2.4 mm in the vertical component. These geodetic values did not form a consistent spatial or temporal pattern of progressive deformation and should be interpreted in relation to the measurement uncertainty.

The analysis of the Dragon’s Den extensometers showed a statistically significant short-term relationship between temperature and measured displacement in the four-hour data. However, the corresponding temperature coefficients were not statistically significant when applied to monthly data covering the entire observation period. This demonstrates that an empirical temperature correction derived from high-resolution data should not automatically be treated as a stable long-term physical model.

The results also confirmed that local telemetric sensors and spatial geodetic measurements provide different but complementary information. Local sensors are sensitive to deformation at selected potentially vulnerable locations, whereas geodetic surveys provide an independent assessment of the broader spatial behavior of the cave. Terrestrial laser scanning further complements these techniques by documenting changes over larger surfaces, although with generally lower point accuracy.

Overall, the monitoring system did not identify significant progressive deformation or structural alarm events during the investigated periods. This conclusion applies to the instrumented locations and available observation intervals. Continued monitoring remains necessary because reliable identification of slow deformation processes requires repeated observations over longer time periods and confirmation of coherent spatial and temporal patterns.

The proposed IoT-based architecture provides a conceptual framework for the integration of multi-sensor measurements, environmental corrections, time-series analysis, and decision-support functions in structural health monitoring. Such integration may improve the continuity of data collection, facilitate the identification of long-term trends and anomalies, and support expert assessment and maintenance planning.

Preservation of historical heritage—particularly sites of the highest significance, as evidenced by their inclusion on the UNESCO World Heritage List—should constitute a priority for developed countries. The implementation of high-resolution sensors capable of quasi-continuous recording for monitoring local rock mass deformations in historic underground structures therefore represents a natural step toward modernizing approaches to their safety management. Tourist attractions that draw large numbers of visitors on a daily basis must be subject to systematic supervision in order to ensure the safety of both visitors and the protected structures themselves.

## Figures and Tables

**Table 1:** Calibration data for crackmeter 459 from Sisgeo laboratory.

Parameter	Notation/Formula	Value	Unit
Linear sensitivity coefficients ^(1)^	A	8.3331 × 10^−3^	mm/digit
	B	−1.7917 × 10^1^	mm
Maximum linear error + U ^(2)^		0.3399	%FS
Final linear reading ^(3)^	Rcl = A × Rmeas + B		mm
Polynomial sensitivity coefficients ^(4)^	A	−2.1381 × 10^−12^	mm/digit^3^
	B	6.1828 × 10^−8^	mm/digit^2^
	C	7.8829 × 10^−3^	mm/digit
	D	−1.7067 × 10^1^	mm
Maximum polynomial error + U ^(2)^		0.0716	%FS
Polynomial MPE		0.3	%FS
Final polynomial reading ^(5)^	Rcp = A × Rmeas^3^ + B × Rmeas^2^ + C × Rmeas + D		mm

**Table 2:** The laboratory and field calibration testing.

Rstd ^(6)^ [mm]	University Laboratory				Dragon’s Den Cave			
	Rmeas ^(7)^ [digit]	U [mm]	Rcl ^(3)^ [mm]	Rcp ^(5)^ [mm]	Rmeas ^(7)^ [digit]	U [mm]	Rcl ^(3)^ [mm]	Rcp ^(5)^ [mm]
0	2131.61	0.01633	−0.1536	−0.0035	2131.780	0.01633	−0.1550	−0.0021
10	3357.39	0.01633	10.0609	10.0150	3356.210	0.01633	10.0510	10.0042
20	4563.16	0.01633	20.1087	19.9881	4561.840	0.01633	20.0972	19.9767
30	5764.57	0.01633	30.1202	30.0195	5763.920	0.01633	30.1141	30.0098
40	6950.10	0.01633	39.9993	39.9886	6951.420	0.01633	40.0103	39.9940
50	8135.46	0.01633	49.8771	50.0048	8136.180	0.01633	49.8830	50.0106
50	8134.38	0.01633	49.8680	49.9956	8135.420	0.01633	49.8767	50.0044
40	6952.07	0.01633	40.0157	40.0052	6953.070	0.01633	40.0244	40.0135
30	5762.40	0.01633	30.1021	30.0013	5761.980	0.01633	30.0979	30.0013
20	4562.53	0.01633	20.1035	19.9829	4563.110	0.01633	20.1080	19.9878
10	3355.99	0.01633	10.0493	10.0035	3355.670	0.01633	10.0465	10.0012
0	2131.92	0.01633	−0.1511	−0.0010	2132.140	0.01633	−0.1520	0.0011

**Table 3:** Multiple linear regression model results.

Sensor	βT [mm/°C]	95% Confidence Interval	*p*-Value for βT	R^2^	Adjusted R^2^	RMSE [mm]
457	−0.01180	−0.01205 to −0.01156	<0.001	0.9397	0.9396	0.00153
458	−0.00885	−0.00932 to −0.00838	3.35 × 10^−160^	0.7902	0.7895	0.00294
459	−0.01251	−0.01265 to −0.01238	<0.001	0.9814	0.9813	0.00228
460	−0.01156	−0.01176 to −0.01136	<0.001	0.9562	0.9561	0.00363

**Table 4:** The regression results for the entire period.

Sensor	βT [mm/°C]	*p*-Value for βT	βtime [mm/year]	*p*-Value for βtime	R^2^	Adjusted R^2^	RMSE [mm]
457	−0.00205	0.2974	−0.00474	0.0996	0.0736	0.0315	0.0209
458	−0.00028	0.8994	0.01820	9.41 × 10^−7^	0.4315	0.4057	0.0237
459	−0.00421	0.0982	0.03601	5.12 × 10^−6^	0.4266	0.4006	0.0516
460	−0.00139	0.5683	0.07406	3.63 × 10^−14^	0.7388	0.7269	0.0502

**Table 5:** Comparison of displacements at selected points of the geodetic network in Series VII and Series VIII.

Point	Series VII: dx [mm]	Series VII: dy [mm]	Series VII: dH [mm]	Series VIII: dx [mm]	Series VIII: dy [mm]	Series VIII: dH [mm]
1A	1.1	1.3	2.4	0.9	0.8	1.9
2A	1.2	1	2.1	1	0.9	1.8
2C	1.4	1.1	2.7	1.4	1	1.8
3A	1.2	0.8	1.8	0.8	1.2	1.5
4A	2.2	2.6	2.8	2	2.6	2.4
4B	1.8	1.8	1.8	1.8	1.6	1.6
5A	1	0.5	1	0.5	0.6	0.8
5B	1.4	0.9	1.4	0.9	1.3	1.2
6A	−0.8	0.2	−0.8	0.2	−0.2	−0.1
10A	0.7	0.4	0.7	0.4	0.2	0.5
11A	0.9	0.5	0.9	0.5	−0.5	0.6

## Data Availability

The datasets presented in this study are not publicly available because they are owned and managed by the museums and site operators described in Section 2. Requests for access should be directed to the corresponding author.
